# Vertebrate gene finding from multiple-species alignments using a two-level strategy

**DOI:** 10.1186/gb-2006-7-s1-s6

**Published:** 2006-08-07

**Authors:** David Carter, Richard Durbin

**Affiliations:** 1 Wellcome Trust Sanger Institute, Wellcome Trust Genome Campus, Hinxton, Cambridge CB10 1SA, UK

## Abstract

**Background:**

One way in which the accuracy of gene structure prediction in vertebrate DNA sequences can be improved is by analyzing alignments with multiple related species, since functional regions of genes tend to be more conserved.

**Results:**

We describe DOGFISH, a vertebrate gene finder consisting of a cleanly separated site classifier and structure predictor. The classifier scores potential splice sites and other features, using sequence alignments between multiple vertebrate species, while the structure predictor hypothesizes coding transcripts by combining these scores using a simple model of gene structure. This also identifies and assigns confidence scores to possible additional exons. Performance is assessed on the ENCODE regions. We predict transcripts and exons across the whole human genome, and identify over 10,000 high confidence new coding exons not in the Ensembl gene set.

**Conclusion:**

We present a practical multiple species gene prediction method. Accuracy improves as additional species, up to at least eight, are introduced. The novel predictions of the whole-genome scan should support efficient experimental verification.

## Background

Gene finding can usefully be viewed as a two-level task. At the lower or local level there is a classification task: one of assigning probability estimates to potential features such as splice sites and coding start and stop sites on the basis of sequence information associated with each potential feature. At the higher or global level, on the other hand, we have a structure-building task: finding the most probable way(s) to combine potential features into exons, transcripts and genes. Classification and structure building are very different tasks, and although a gene finder can be based on a single formalism, such as hidden Markov models (HMMs) [1,2], there is no reason to assume that the same technique will be optimal for both tasks. Although HMMs seem to offer a good basis for structure building, they impose independence assumptions that are not particularly well suited to feature classification; formalisms such as neural networks [3,4], maximum entropy modeling [5], Bayesian networks [6-8], support vector machines [9-11] and relevance vector machines (RVMs) [12-14] provide alternative approaches with potential benefits.

Gene finders have conventionally analyzed a single sequence [2,15-17] or, more recently, alignments between sequences for two species [18-25]. In the past year or two, gene finders processing alignments of more than two species have begun to appear [26-31]. In principle at least, the additional information provided by extra species should lead to improved predictions, but it is far from trivial to extend existing formalisms to make the best use of it.

In parallel with systems processing only genomic data, gene finders have been developed to use expressed sequence tag (EST), cDNA and protein sequences [32-36]; these can achieve better overall accuracy than systems using multiple-species alignments, but they are effective only where the sequences in question have been detected.

The gene finder described in this paper, DOGFISH (for 'detection of genomic features in sequence homologies'), is based on the above observations. It predicts gene structures in the sequence for a target species based on alignments with one or more informant species. At the global, structure-building level it employs a fairly conventional HMM. Its two main novelties lie at the local, classification level. At this level, it analyses multiple-species alignments (of eight species in the work reported here), passing the results up to the HMM for structure building. In this way, it avoids having to deal with the complexities of multiple-species alignments and the HMM formalism in the same tightly coupled framework. To do the classification, it uses a cascade of relevance vector machines to derive a single probability estimate from many thousands of individual scores based on particular aspects of the aligned sequences around a feature of interest. The HMM sees only the predictions of the classifier, not the genomic sequences or alignments, resulting in some useful simplifications.

## Results and discussion

In this section, we present results first for classification of individual splice sites and start and stop codons, and then for HMM-based gene finding on the ENCODE test regions using the outputs of the classifier.

### Classifier results

**Figure 1:**
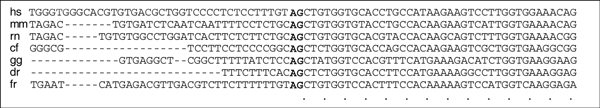
Alignment for a coding splice acceptor site. The figure shows the central part of a typical alignment window used by the classifier component of DOGFISH. Codon boundaries on the exon side of the splice site are indicated with dots. This site has an alignment with all species except frog: hs; *Homo sapiens*: mm; *Mus musculus*: rn; *Rattus norvegicus*: cf; *Canis familiaris*: gg; *Gallus gallus*: dr; *Danio rerio*: fr; *Fugu rubripes*. The AG dinucleotide for the acceptor site itself is shown in bold.

As explained in more detail in the Materials and methods section, DOGFISH's classifier consists of two main components, which adopt respectively a 'vertical' and a 'horizontal' view of alignments of multiple species around each feature of interest (see Figure 1 for an example alignment). The vertical component applies a separate evolutionary model to each column in an alignment, explicitly modeling mutations but taking only very limited account of the context in which the column occurs. The horizontal model is complementary: it uses Markov models and nucleotide tuple frequencies to assess the aligned sequence for each species as a possible instance of the feature under consideration without reference to the other species, and then combines the results to produce a single estimate. Thus, in contrast to the vertical model, it analyses context as thoroughly as possible but ignores mutations. Since both kinds of information are important, one might expect each component to perform well on its own, and a combination of the two to do better still.

We trained DOGFISH to detect genes in the human genomic sequence on the basis of the University of California, Santa Cruz (UCSC) MultiZ alignments [37] with seven other species. We used the multi-way alignments with mouse, rat, dog, chicken, zebrafish and fugu, discarding chimp from the original set because it did not improve results, and adding in the separately available human-frog pairwise alignments. All sequences were soft repeat masked using RepeatMasker [38]. The classifier was trained and evaluated using all the Vega annotations for human (nine chromosomes, downloaded August 2005), excluding those for all 44 ENCODE regions and for positions 100 M to 110 M of chromosome 9, a region with typical gene density that we used for various tuning purposes. We did not use the 18-species ENCODE comparative sequences [39], which were only available for the ENCODE regions, covering 1% of the human genome, for two reasons. Firstly, this quantity of sequence would not be enough to train fully the thousands of parameters in the classifier. Secondly, we wanted to run the system on the whole human genome, for which the UCSC alignments were the most comprehensive available.

At the local level, DOGFISH assigns a probability estimate to every potential splice site, start codon and stop codon in a genomic region to be analyzed and, for splice sites only, a probability distribution over the possible coding phases. A potential splice site is defined here as any AG or GT dinucleotide; GC splice donors and U12 splice sites are too rare to be accurately detected. In what follows, by a 'true' acceptor site we mean any AG splice site, while a 'decoy' is an AG that is not a splice site. True and decoy donor splice sites and start and stop codons are defined similarly.

We evaluated a number of variants of the classifier on a specially constructed 'challenging' set of candidate sites. The probability of including a site in this set, irrespective of whether true or decoy, was a strongly increasing function of the score assigned to it by a first version of the classifier that was itself trained on randomly selected sites. Such a challenging set is necessary to achieve clearly distinct performance figures; if sites are randomly selected from the genome, the classification task is too easy, at least for splice sites, and many versions of the classifier score close to 100%.

#### Comparing classifier components

We evaluated performance using the horizontal component alone, the vertical component alone, and both together. As well as the scores derived from each of the horizontal and/or vertical components, we used one further value in all the experiments. This was derived from a simple 'presence' component that just returns a score depending on the set of species aligned to a site, irrespective of the content of the alignment; thus, a site that aligns with many informants is likely to score higher. This favors true sites because the true splice sites in our challenging set align with locations in an average of 5.6 out of our seven informant genomes, compared to 2.0 for decoys, while for start and stop sites the corresponding figures are 3.8 for true sites and 1.6 for decoys.

We also evaluated the full classifier against all potential sites in the roughly 21.5 Mb of the 31 ENCODE test regions. In genomic regions, decoy sites were thousands of times more numerous than true ones, rather than just a few times as in our main evaluation set. This serves as a 'reality check' that our main set, despite its challenging nature, is not artificially easy. For comparison, we also evaluated the site estimates output by the full gene finder; these values are based partly on the classifier estimates but also on the availability of nearby sites to make up legal gene structures.

F-score and receiver operating characteristic (ROC) error values are shown in Table 1 for each condition. These results can be summarized as follows. Firstly, start and stop codons are much harder to detect than splice sites. Secondly, for splice sites, presence scores alone are much better than random: the F scores in the 'Presence' line of the table are well over the small percentage of true sites in the evaluation set, which would be the F scores expected from a random-choice strategy. Thirdly, adding either the vertical or the horizontal component improves performance markedly over using the presence component alone. Fourthly, for splice sites, the horizontal component alone is better than the vertical component alone. Fifthly, using both the horizontal and the vertical component is consistently, but only slightly, better than using the horizontal alone. Sixthly, classifier results on the ENCODE regions confirm that performance is good on whole genomic regions, where decoys outnumber true sites by thousands to one. (The simultaneous decrease in both F score and ROC error rate is a consequence of these regions having far more, but on average easier, decoys than the main test set; see Materials and methods.) Finally, not surprisingly, the full gene finder is much more accurate than the classifier alone on the ENCODE regions.

#### Analysis of classification errors

The errors in classification on the challenging test set are broken down by site type in Table 2. For this table, we set acceptance thresholds so that false positives balance false negatives. (We treat a decoy as 'coding' not only if it falls within a coding region of the genome but also if it is within 50 bases of a coding region. In the latter case, it will generally have 50 or more coding positions within the 200-nucleotide region described in Materials and methods, making it in that regard more similar to a true coding site, which usually has 100, than to a true non-coding site, which usually has none.)

Not surprisingly, the figures indicate that non-coding splice sites are harder to detect (have a higher error rate) than coding ones. However, we were initially surprised that intergenic splice site decoys (which are by definition non-coding) should have a much higher error rate than intragenic non-coding or even coding ones. This could be due either to suppression of non-functional splice sites inside transcripts or to non-annotated exons outside annotated transcripts. We found no evidence of suppression (decoys inside and outside transcripts were similarly distributed) but we did find evidence for unannotated exons.

If substantial numbers of exons are present in a region, one would expect high-scoring candidate acceptor (A) sites to alternate with high-scoring donors (D) more often than chance would predict. Therefore, we looked at the highest-scoring *N *acceptor candidates and the highest-scoring *N *donors, for various values of *N*. If no exons are present, we would expect neighboring AD and DA pairs on the same strand to occur no more often than AAs or DDs. However, if there are exons, then as *N *rises, we expect Δ, the excess of ADs and DAs over AAs and DDs, to rise as genuine splice site pairs enter the set, then to fall again as the pattern is destroyed by lower-scoring, mostly decoy sites.

We looked at how Δ varied with *N *on human chromosome 13. This chromosome was selected because, in proportion to its length, it had contributed the smallest number of sites to the top-scoring 2% of intergenic decoys to the test set and, therefore, seemed likely to contain the fewest unannotated exons. Even on this chromosome, we found Δ rising to a highly significant level and then falling again, as predicted. At maximum, we found a total of 2,062 AD and DA pairs in the chromosome 13 intergenic regions, compared to 1,712 AA and DD pairs, giving Δ = 350. The corresponding Δ value for intragenic regions was 4,773 - 1,658 = 3,115. The Vega annotation of chromosome 13 contains about 3,000 internal (bounded by an acceptor and a donor) exons, which would suggest there are around 3,000 × 350/3,115 = 337 exons still be to be annotated. We return to the implications of this later.

#### Splice site phase determination

We have seen that using the vertical component in addition to the horizontal one does not improve splice site detection by more than a small amount. However, this is not the case for the task of determining splice site phases. For coding splice sites, the error rate (percent incorrect) for the various combinations is given in Table 3; we take a prediction as correct if the true phase is the one assigned the highest probability. These results show that for phase determination, the vertical component is superior to the horizontal. This would appear to be because the vertical component explicitly looks for patterns of amino acid conservation, which are a more powerful indicator of phase than the per-species nucleotide preferences detected by the horizontal component. However, using both vertical and horizontal is much better than using vertical alone, suggesting that the horizontal component, with its wider view of context, is picking up phase-indicating contextual effects wider than individual codons, even though it does not compare sequences so is blind to patterns of mutation.

#### The effect of additional species

Finally, we tested one of the assumptions behind this work, that the more informant species are used, the better the classifier works. We evaluated the configuration of the system containing the species-presence and horizontal components of the classifier trained on challenging data; this is almost as accurate as the full system in classifying splice sites. We made available one species (human), two (human and mouse), four (the mammals) and all eight; Table 4 shows the results. As expected, the greatest gain comes from the first additional species, mouse. However, more gains are apparent as further species are added, with non-mammal species apparently just as useful overall as additional mammals.

### Gene finder results

We combined the classification results into gene structures using an HMM as described in Materials and methods. In the evaluation here, we focus on exon performance as the primary indicator.

Table 5 gives sensitivity and specificity results at the nucleotide, exon and transcript level on the 31 ENCODE testing regions, for DOGFISH-1, the version available at the time of the ENCODE competitive evaluation in May 2005, and for DOGFISH-2, the current version. Although the latter version was developed after the detailed annotations of the testing regions were released, no nucleotide sequences, alignments or annotations for any of these regions were used in any way in developing any version of DOGFISH.

DOGFISH-2, the current version of the system, is described throughout this paper. The most important differences between DOGFISH-1 and DOGFISH-2 are as follows. Firstly, although DOGFISH-1 constructed coding-phase-specific models within the horizontal and vertical components, the RVM cascade did not maintain separate per-phase hypotheses during its later data reduction. This both decreased the accuracy of its estimates and meant it was unable to pass phase information on to the HMM. Secondly, DOGFISH-1's HMM component was less sophisticated than that of DOGFISH-2, and in particular did not use N-best lists [2] (see Materials and methods) to mitigate the negative effects of using exon and intron length penalties. Thirdly, the training set used for DOGFISH-1's classifier was not constructed systematically to include difficult decoys and, therefore, the classifier was less well-matched to the needs of the gene finder.

#### Error analysis

The gene-finding results for both DOGFISH-1 and DOGFISH-2 are derived from the single best-scoring HMM path; thus only one transcript per gene is predicted, a bias that is reflected in sensitivity scores being rather lower than specificity. In fact, the excess of false-negative over false-positive exon detection errors made by DOGFISH-2 on the ENCODE test set is almost exactly equal to the number of alternative exons in the reference annotation; these account for half of all exon errors.

The next most important source of errors is the classifier's poorer performance on start and stop codons than on splice sites. The overall exon sensitivity of 63.68% in fact breaks down to around 73% for internal exons and only 37% for external (initial and terminal) ones, while specificity (84.90% overall) is 87% for internal exons and 73% for external. This difference directly accounts for about a quarter of all the exon errors, and has an additional knock-on effect in the form of increased numbers of errors in internal exons adjacent to external ones, accounting for a further 20% of the errors. Most of the final 5% of errors can be traced to imperfect classifier estimates on splice sites.

This analysis suggests a number of ways in which DOGFISH could be improved. Firstly, by explictly modeling splicing signals not currently handled, such as enhancers and repressors; this could be done by applying independently derived information to train weight matrices for such signals, which can be longer than the six-nucleotide patterns processed by the current method. Secondly, by an explicit treatment of the specific characteristics of alternative exons [40]; including high-scoring exons not on the HMM's best path as suggested in [41] did not work well. Thirdly, by better modeling of untranslated regions [7,28]. Fourthly, by using alignments with more informant species, both closely related and more distant. Fifthly, by improving accuracy on start and stop codons.

Of these, there is reason to hope for good progress from applying variants of DOGFISH's existing machinery to the first four problems; but we have already devoted substantial effort to the last issue, start and stop codons, and it is not clear to us how much better accuracy could be obtained for these features. The difficulty seems to be that despite the known consensi around these sites, interspecies conservation is not as strong as for splice sites and so a multiple-alignment based method cannot predict them as accurately.

#### Exon probability estimates

**Figure 2:**
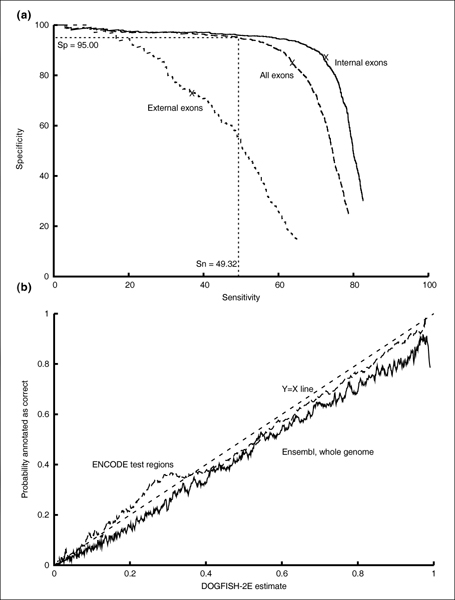
DOGFISH-2E results. (**a**) Sensitivity and specificity for DOGFISH-2E output. The figure shows plots for specificity against specificity on the ENCODE test regions as the acceptance probability threshold is varied for internal exons, external (initial and terminal) exons, and all exons together. 'X' is used to mark the DOGFISH-2 sensitivity and specificity values, and the specificity value of 95% for almost 50% sensitivity is highlighted. (**b**) Probability of annotation as a function of DOGFISH-2E estimate. The figure shows DOGFISH-2E probability estimates on the *x *axis and, on the *y *axis, the probability that a site a DOGFISH-2E estimate of the given magnitude is annotated in ENCODE and Ensembl, respectively. The Y = X line is shown for comparison.

The gene finder HMM assigns a score to every candidate site and exon. Using these scores, we trained separate relevance vector machines (RVMs) for initial, internal and terminal exons to estimate the probability of correctness of each candidate coding exon. By setting the threshold for acceptance, we were able to trade off sensitivity against specificity. We call this version of the system DOGFISH-2E, since it predicts individual exons with no requirement that they make up correct transcripts; this could indicate additional exons incompatible with the most likely gene structure, and also allows low-scoring exons (even when on the best path) to be discarded. Figure 2a shows the behavior on the ENCODE test regions for internal exons, external exons (initial and terminal individually show similar behavior) and all exons together. The points corresponding to DOGFISH-2 are shown there as crosses; note also that close to 50% of all exons are predicted with specificity 95% or better.

#### Whole-genome scan

We ran DOGFISH-2E over the whole human genome (excluding chromosome Y because of its overlap with X), estimating probabilities for over 1.3 million candidate exons, and looked at how these estimates correlated with whether each exon was among the 181,475 coding exons in the Ensembl database (downloaded 9th November 2005). We found that the probability of an exon being present in Ensembl was very well modeled by its DOGFISH-2E estimate multiplied by 0.889 (compare Ensembl's 0.775 sensitivity against the ENCODE annotations; see companion paper in this supplement). For DOGFISH-2E on the ENCODE test data, the corresponding factor was 1.001, though the relationship was less linear (Figure 2b). It seems likely from the difference between the factors that substantial numbers of exons are missing from Ensembl.

DOGFISH-2E assigns an estimate of 0.95 or greater to 99,369 exons over the whole genome. On the ENCODE test data, 95.9% of exons scoring over this threshold are annotated as correct; thus, it seems reasonable to assume that 0.959 × 99,369 = 95,295 of the whole-genome predictions are correct. Of the 99,369, only 88,385 are annotated in Ensembl as coding exons, with 10,984 either absent altogether or, in a minority (15%) of cases, annotated as non-coding. Even if we assume that all of the 88,385 are correct, we are left with an expected 95,295 - 88,385 = 6,910 correct predictions among the 10,984 additional ones, giving a specificity of 62.9%. Adding 6,910 new coding exons to Ensembl's existing total of 181,475 would increase the number by 3.8%.

These results, together with the pattern of alternation of high-scoring 'decoy' acceptor and donor splice sites in regions annotated as intergenic in Vega, lead us to conclude it would be fruitful to use high-scoring DOGFISH-2E predictions to guide experiments searching for new coding exons. It would also be interesting to investigate how far these 'missing' exons overlap with existing EST data and with so-called transfrags [42].

## Conclusion

Distinguishing two levels of the task of gene finding allows separate strategies to be applied at each level, allowing us to make good use of the information present in multiple alignments without the system becoming unmanageably complex. The current accuracy of DOGFISH is comparable to that of the best published gene finders that use multiple-species alignments (see other papers in this supplement), confirming that a two-level approach can yield good results.

Perhaps surprisingly, vertical (evolutionary) models do not appear to offer much advantage over combining the results of horizontal ones when it is a matter of distinguishing true sites from decoys; however, they are useful for determining phase, a task that is important for guiding the gene finder, since a phase mismatch can help rule out an otherwise promising exon.

The strategy of using multiple species pays off: we have demonstrated that the more species are used, the more accurately splice sites can be detected. It remains to be verified whether this effect will continue to apply if more than eight species, or different species, are used, but Table 4 does not suggest that saturation has been reached. Furthermore, adding more closely related informants as their genomes become available should also improve performance, since 3.4% of confirmed coding splice sites in our data set have no alignments at all, and a further 3.5% only align to one other species.

Three useful resources arise from this work. The first is the challenging data set used to train Classifier Two, which we offer for use for training and testing both single- and multiple-species feature classifiers. The second is the single-species subpart of the horizontal component, which is a strong single-sequence classifier in itself. The third is a set of predictions of splice sites, exons and genes obtained by running DOGFISH over the whole human genome, which will enable experimental effort to be concentrated on predictions that are not part of known genes; we estimate that if the highest-scoring 50% of these extra predictions are selected, over 60% of them will be correct.

## Materials and methods

In this section, we devote most attention to DOGFISH's classifier, which contains most of the novel aspects of the system. We finish with a description of the structure-building HMM, focusing on the way it uses classifier outputs and the respects in which it differs from conventional HMM technology.

### Classification methods

The main mechanism that DOGFISH uses in its classifier is the Biojava [43] implementation of the RVM [12,14], a robust and accurate new classification technology that dispenses with many of the independence assumptions inherent in HMMs. An RVM is a trainable device for mapping any number of input scores (which may or may not themselves represent probabilities) to a single output probability. In contrast to most other classification methods, when the mapping is trained, a few inputs typically receive high weights (are viewed as 'relevant'), a few more get low ones, and many are assigned a weight of zero, on the basis that they do not offer any further useful information once the other inputs, with which they may be correlated, have been taken into account. The tendency of RVMs presented with many inputs to select only a few of them as relevant leads to good robustness, greater transparency than some alternative techniques, and some efficiency gains because the values of zero-weighted inputs do not need to be calculated.

DOGFISH applies a cascade of RVMs to carry out a stage by stage reduction of many thousands of scores, each derived from one small facet of an alignment around a site of interest, to a single estimate of the probability that the site is a true instance of a particular feature such as an acceptor splice site.

DOGFISH classifies a feature by looking at a 200-nucleotide window centered on it. Each column of the window contains a target-species nucleotide and, for each informant species, either a gap character or a nucleotide from that species. The window is much wider than the known consensus of a dozen or so base-pairs around splice sites; however, this choice makes it possible to detect not only these consensi but also coding phases and transitions between introns and exons and between non-coding and coding regions, both of which are marked by distinctive patterns of conservation and divergence in the alignments. Doing this removes most of the need for an explicit model of coding sequence in the HMM, which is able as a consequence to avoid looking at nucleotides altogether and work simply on the classifier output scores.

The inner 78 positions of a classifier window, for a typical phase-zero acceptor site, are shown in Figure 1. Sequences from seven species are aligned here, with species identifiers shown to the left; the top one is the human sequence, and the frog sequence is missing. The AG dinucleotide at the site itself is shown in bold, and codon boundaries are indicated by dots under the alignment. Characteristically for this type of site, we see much better alignment on the exon (downstream) side than the intron side; a polypyrimidine tract just upstream of the site, clearly present in all species but with poor inter-species alignment at the nucleotide level; and, on the exon side, at least close to the splice site, more mutations in codon-final positions. The classifier uses all this information not only to distinguish true sites from decoys, but also, for the case of splice sites, to determine coding phase.

### 'Vertical' and 'horizontal' perspectives

There are many ways in which a classifier could be trained on such a data structure, but two are clearly worth pursuing. As discussed briefly above, we call them vertical and horizontal approaches according to which dimension of the window they treat as primary.

In the vertical approach, we look primarily at the columns of the window, each of which contains the target-species nucleotide at a particular offset from the (candidate) site in question and its alignment, if any, with each informant species. We apply offset-dependent evolutionary models to derive a score for each column having arisen at that offset from a feature of the type under consideration (for example, 17 bases upstream of a phase-zero splice donor). We then, secondarily, look at the horizontal dimension, combining the per-offset scores resulting from the primary step into a single estimate.

By contrast, in the horizontal approach, we first treat the sequence for each species as a potential instance of the feature in question and derive an estimate of the probability that it is indeed one. We then, secondarily, combine these species-specific estimates together (making suitable allowance for one or more species being absent altogether) into a single estimate.

Each approach has its strengths and weaknesses. The vertical approach involves an explicit treatment of mutation at a given position but, because of the complexities of evolutionary models, it can take only limited account of contextual influences between neighboring positions [29,44,45]. In contrast, the strength of the horizontal approach is a thorough treatment of just these influences, at the price of ignoring the relationships between aligned nucleotides. The complementary nature of these two approaches means there is reason to hope that a combined approach will do better than either one on its own.

We accordingly combine the two components on an equal basis, in the following sense. For each window to be evaluated, the horizontal component makes eight predictions (one for each available species) that are then combined into a single one. We therefore implemented the vertical component also to make eight predictions by dividing the 200-nucleotide window into 8 subwindows of 25 bases, and combining each set of 25 column-specific scores to produce a single value. We then combine the 16 resulting values (one horizontal for each species, and one vertical for each 25 base-pair subwindow) into a single estimate.

For our vertical component, we use the PAL phylogenetic analysis package [46], selecting the generalized time-reversible model of mutation [47]. We train separate sets of models on sets of true and decoy candidate sites and on sites of different coding phases. We also distinguish intragenic from intergenic decoy sites, giving us nine 'site types' for acceptors (phases zero, one and two true sites, non-coding true, phases zero, one and two decoys, and two types of non-coding decoy), nine for donors, and six each for starts and stops (since true instances can only be phase zero). Within each 25 base-pair subwindow, we divide the training data differently depending on whether that subwindow represents a coding or non-coding region in the target species. For a 25 base-pair non-coding region, we train each offset with a separate model, yielding 25 models. For a coding region, we train separately for each codon position of each amino acid or stop codon, yielding 3 × (20 + 1) = 63 models. Thus, in total, over all subwindows and site types, we trained over 2,700 evolutionary models for each kind of splice site and over 1,800 for both coding starts and stops. This was possible because of the tens of thousands of training examples available to us, each containing information at every offset.

Because PAL models only mutations and not gaps, we included in the vertical model a simple gap model that applied an RVM to the counts of gaps, and ungapped runs of nucleotides, of particular lengths in particular parts of the window. For example, one such feature would be number of gaps of length 4 to 15 starting (in any species) at an offset between 0 and 25 to the right of the center of the window. In subsequent processing, the estimate derived from the gap model was treated just like each of the eight estimates for 25-base subwindows.

For the horizontal component, we again train separate sets of models for each site type. We analyze each sequence in two ways. Firstly, we estimate the likelihood of each nucleotide using position-specific weight matrices [1], using a context length of up to six nucleotides; smoothing is achieved by only using a longer context when the distribution of its predicted target nucleotide is significantly different on the training data from that given by a shorter context. Secondly, we look for the words of length six or less whose frequency of occurrence over given parts of the window varied most between training sets. For example, the triplet TCT is much more common in the 20 bases upstream of true acceptor sites than of decoy AGs because of the presence of the polypyrimidine tract in true acceptors. To detect coding biases, we counted both overall occurrences of this type and occurrences starting at offsets differing by a multiple of three.

### Combining estimates using relevance vector machines

Each component thus yields several hundred different scores on each candidate site for each hypothesized site type. We reduce these to the final true-site and phase probability estimates for a site as follows.

First, we considered each pair of possible types for the site, for example, phase-zero true with phase-two decoy, taking logs of ratios of corresponding estimates in both the horizontal and vertical components. For each pair, we train a RVM on the scores from the horizontal component (using target-species sequences), and one RVM for each of the subwindows in the vertical component. Each of these RVMs selects anything from a handful of its inputs to nearly all of them as 'relevant', and maps from those scores to a single output. Site types are considered in pairs rather than all together because an efficient approximation for the optimization process involved in training the RVM is only known for the case of two classes, not multiple ones.

By this stage, for each pair, we have eight RVM output scores from the horizontal component (one for each species present in the alignment, with suitable trained defaults used where species were absent), and nine from the vertical component (one for each subwindow and one for the gap model). Next, we train another RVM to combine these scores (plus that of the 'species-presence' component) into a single estimate for the probability that the given instance represents one of our current pair of site types rather than the other.

Each kind of splice site, as we have seen, has nine types, yielding 9 × 8/2 = 36 different pairs, and coding starts and stops have six, yielding 15 pairs. Our next step is thus to train a further RVM to make the true versus decoy distinction on the basis of all decoy-and-true site type pairs. For splice sites, we also train one to predict the probability of each phase among true sites on the basis of all true-true pairs. The outputs of the phase RVMs are then normalized so that they sum to one in the probability domain.

Finally, for reasons explained below, we run two separate instances of the classifier trained on two different data sets, and average their results together; we could have trained RVMs to do this step, too, but we found that performance was quite insensitive to the weights used.

**Figure 3:**
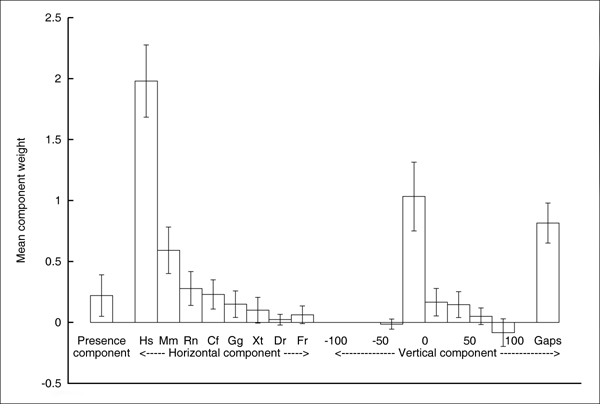
Mean RVM weights for horizontal and vertical component inputs. The figure shows the means, with *p *= 0.05 two-tail error bars, for weights assigned to inputs by acceptor site-type-pair RVMs in Classifier Two, averaging over all 20 pairings of decoy with true site types. The presence component has a single score. Two-letter abbreviations are used for the species-specific scores output by the horizontal component, while the vertical-component quantities are for eight 25 base-pair subregions (only six of which ever get non-zero scores) with one gap score. Species abbreviations are as in Figure 1.

Figure 3 illustrates one stage of the data-reduction process, showing how one presence, eight horizontal and nine vertical scores are weighted. The values given are means over all 20 (5 decoy types by 4 true) acceptor site RVMs for Classifier Two, with *p *= 0.05 error bars on the means. Each RVM input is separately prenormalized to have a standard deviation of one, so that the weights are directly comparable. It can be seen that the weights given to horizontal-component scores decrease with evolutionary distance from human. The vertical component gives a lot of weight to its gap model and to the subregion from 0 to 25 bases upstream of the AG, and some weight to those 0 to 25 and 25 to 50 downstream, but effectively none to any others, since their weights are either always zero or are on average indistinguishable from zero. The presence component makes a positive but small contribution.

### Rational choice of training data

Choosing appropriate training data for the local level of DOGFISH is an important and non-trivial issue, because there are thousands of times more decoy sites in a genome (in the sense of specific di- and trinucleotides) than true ones. Training a classifier with many parameters usually gives best results with many thousands of true sites, which implies using a significant portion of the genome, containing many millions of decoys. Processing all those decoys in training may not be practically feasible; and even if it is, doing so may well, as pointed out in [48], result in a classifier that rejects every item.

The imbalance can be reduced or even eliminated by procedures such as random sampling of decoys [6] or only considering regions known to be relatively rich in true instances, such as the coding extents of genes ([49], resulting in a decoy-to-true ratio of around 100). However, random sampling is likely to leave the classifier somewhat undertrained on the more difficult decoys, only a few of which will be selected for training; and annotation-based region selection will systematically exclude whole classes of decoys, many of which may be difficult ones (compare the large proportion of intergenic false positives in Table 2). Both procedures represent a partial mismatch with the requirements of the gene finder, which has to process whole genomic regions and is especially likely to be misled by poor classifier estimates on the more challenging decoys. Therefore, although we do need the classifier to reliably recognize the easier decoys that form the vast majority of the sites it will encounter, we also need it to be well-trained on challenging ones.

We therefore train and run two versions of the classifier, and give the gene finder the average of their estimates. Classifier One is trained using a large set of true sites and randomly sampled decoys. The training set for Classifier Two is constructed by running the classifier one and the gene finder over the whole Vega portion of the genome. To do this in reasonable time, we run Classifier One in a 'lite' mode in which the horizontal component only examines the target (human) sequence, and the vertical component is replaced by a much simpler one based on counting occurrences of codons and amino acids in different site types.

We then create a training set for the Classifier Two by a highly non-uniform random selection process, favoring high-scoring sites from the output of the first-pass HMM, irrespective of whether they are true and decoy, but without excluding low-scoring ones altogether. Crucially, this selection process does not rely on any form of annotation. The result is a set consisting of nearly all the true sites that have a reasonable chance of being detected by DOGFISH, and several times as many decoys, most of which are challenging ones. Around 20% of true splice sites and 65% of true start and stop codons are omitted, along with the vast majority of decoys, because they score low as a result of aligning with few species and/or not reflecting the consensus sequence well.

Training Classifier Two on this set has the effect of tuning it to the hardest kinds of decisions that the second-pass HMM will ask it to make. Furthermore, we believe that this training set is of interest in its own right as a challenging testbed for genomic feature classification, since it is enriched for difficult (that is, realistic, from a gene-finding perspective) decoy cases rather than being made artificially easy by being enriched for true sites on the basis of existing annotations.

To train each classifier instance, we first divided the data into 10 roughly equal-sized portions, P1 to P10. P1 and P2 were used to train the underlying horizontal and vertical models (Markov, word-based and evolutionary); P3 to P6 to train the intra-component RVMs; P7 and P8 to train the site-type-pair RVMs; P9 to train the RVMs to produce the final estimates; and P10 (taken from challenging, second-pass data set for both classifier instances, not just for Classifier Two) for evaluation. The classifier results given in this paper are for two evaluation runs, in one of which P9 and P10 were exchanged. The gene-finding results instead used both P9 and P10 together to train the final RVMs; there was no need to hold either of them out, as the entire data set under discussion here is disjoint from all the ENCODE regions.

To avoid the training and evaluation sets being too similar to each other and thereby artificially boosting the accuracy scores, we allocated sites to portions not at random but so as to ensure that as far as possible, paralogs were allocated to the same portion. First, all sites (true and decoy) from within the same gene were put in the same portion. Second, genes were clustered so that as far as possible, two genes that both (partially) aligned to the same piece of informant sequence were also put in the same portion.

### The global level: structure building using HMMs

Most of the complexity of DOGFISH is, as we have seen, located in its local-level classifier, allowing the global-level HMM component to be relatively simple. The system works as follows. Every potential splice site and start and stop codon on both strands in the target sequence is handed to the classifier, which, as we have seen, returns an estimate of the probability that the site is a true instance of the feature in question, accompanied, for splice sites only, by a probability distribution among the four possible coding phases (zero, one, two and non-coding) conditioned on the site being a true one. The HMM sees only these estimates, not the DNA sequences themselves, and searches for the best-scoring combinations of sites that are consistent with (its model of) the structure of protein-coding genes. Before this search is carried out, the site scores undergo linear transformations, with different parameters for splice sites and for start/stop sites; parameters for these transformations were optimized on the 13 ENCODE training regions for evaluation on the 31 testing regions.

The HMM's topology imposes several simplifications on biological reality. Firstly, no attempt is made to model transcription start sites and polyadenylation sites. Instead, a gene starts either with a start codon (for the case where coding starts in the first exon) or with a non-coding splice donor (the end of the first exon where coding starts in some later exon). Similarly, it ends with either a stop codon or a non-coding splice acceptor. Secondly, non-coding transcripts are excluded for the same reason. Thirdly, genes with a single coding exon are handled, but are not treated specially despite evidence [50] that they should be: such genes often arise from reverse transcription of mature mRNAs, so that their single exon tends to be as long as several exons in the more common kinds of genes. As a result, few are predicted. Fourthly, no provision is made for overlapping or embedded genes, on either the same or opposite strands, although alternative paths through the lattice can be pulled out once the HMM has run. Fifthly, no provision is made for start and stop codons interrupted by introns, largely because of the difficulties of training the classifier on sufficient numbers of these relatively rare cases. Sixthly, as stated earlier, only AG acceptor sites and GT donors are considered, for similar reasons.

The first of these simplifications is applied because transcription start sites and polyadenylation sites are notoriously hard to model accurately and in most cases are not even known precisely. Each of the other simplifications makes the overall model simpler, excluding various rare and, therefore, hard-to-train cases; we believe that these decisions make an overall positive contribution to accuracy by ruling out many false positives, at the admitted cost of also excluding a relatively small number of correct structures.

The only respect in which DOGFISH's HMM departs from the basic technology is that it explicitly models the observed distributions of exon and intron lengths, penalizing very short introns and exons. These penalties can be applied only to complete hypothesized exons and introns, not to partial ones, with the consequence that the algorithm is no longer quite sound: the overall least-cost path is no longer guaranteed to be found. To mitigate this effect, we maintain at each position a N-best list [2] of the best few path continuations in each direction, rather than just the best one. We have found *N *= 5 maintains reasonable efficiency while excluding few if any correct and (theoretically) highest-probability paths. Accuracy is much improved overall by modeling lengths; for example, if they are not modeled, many more very short exons and introns (lengths less than 20 and 50 nucleotides, respectively) are accepted than really occur.

The DOGFISH-2E exon probability estimates were derived by training three separate RVMs, for initial, internal and terminal exon candidates, respectively. The inputs to each RVM were the scores assigned by the gene finder to the sites and each end of the exon; the log of the length of the exon; and the 'competition score', the difference between the HMM score for the exon itself and that of the best-scoring overlapping exon. The competition score is positive for exons on the best-scoring path and negative for all others; the RVMs for internal and terminal exons used it almost to the exclusion of all the other inputs, while the initial-exon RVM mainly favored the minimum of the two end-site scores. For evaluating DOGFISH-2E on ENCODE test data (Figure 2a), we trained only on the ENCODE training regions, while for the whole-genome scan we used RVMs trained on all the ENCODE data; the resulting differences appeared to be minimal.

### Software

The DOGFISH comparative gene finder software and its predictions on the human genome are available under the GNU public license at [51].

## Authors' contributions

D.C. and R.D. designed the experiments and completed this paper; D.C. wrote the software, carried out the experiments and wrote the first draft of the paper.

## Figures and Tables

**Table 1:** Prediction accuracies for vertical and horizontal components

	Acceptors	Donors	Starts	Stops
Train set size	204,021	221,421	7,571	25,071
Eval set size	52,605	57,179	1,805	6,162
%True sites	14.05	13.01	16.68	8.08
**F scores (%)**				
Presence	52.72	48.77	39.70	34.64
Vertical	82.01	81.00	55.70	49.25
Horizontal	84.36	84.43	57.01	48.22
Both	84.86	84.60	58.22	49.60
ENCODE Cl	63.18	65.86	27.44	14.67
ENCODE GF	80.23	81.38	42.47	50.49
**100-ROC (%)**				
Presence	12.41	12.66	20.62	23.98
Vertical	2.46	2.52	14.49	12.76
Horizontal	1.81	1.58	12.48	11.77
Both	1.74	1.54	10.41	10.90
ENCODE Cl	0.99	0.61	9.14	10.49

The table shows the F score (geometric mean of sensitivity and specificity, which are close to each other) for various classifier components. The test set for the presence, vertical, horizontal and 'both' conditions is 'challenging' data; we show results for a mixture of the classifiers trained on challenging and randomly selected data. The 'ENCODE Cl' and 'ENCODE GF' lines are for the 31 ENCODE test regions, using classifier scores and gene-finder scores, respectively. The table also shows the 100%-ROC (receiver operating characteristic) error value for each condition. This error value is the probability that if a true instance and a decoy are selected at random, the classifier will give the decoy a higher score than the true instance.

**Table 2:** Error rates broken down by site type

	Acceptors	Donors	Starts	Stops
**True sites, false reject percentages**				
Overall	4.45	4.08	17.61	15.86
Coding	4.34	3.88	17.61	15.86
Non-coding	7.48	10.00	NA	NA
**Decoy sites, false accept percentages**				
Overall	4.45	4.08	17.35	15.84
Coding	2.34	1.08	23.19	2.92
Non-coding intra	2.54	2.21	12.17	16.58
Non-coding inter	8.00	8.31	18.05	16.07

The table shows the proportion (in the challenging test set) of various site types that received an incorrect classification. The classification threshold is adjusted to achieve roughly equal proportions of false positives and false negatives. NA: not applicable.

**Table 3:** Phase prediction error rates on coding splice sites

	Acceptors	Donors
Vertical	3.84	3.02
Horizontal	5.17	4.79
Both	1.99	1.60

The table shows the percentage (in the challenging test set) of coding splice sites for which the coding phase that was assigned the highest probability was not the annotated phase.

**Table 4:** Prediction accuracies for different numbers of species

	Acceptors	Donors	Starts	Stops
Train set size	204,021	221,421	7,571	25,071
Eval set size	52,605	57,179	1,805	6,162
**F scores (%)**				
Human only	66.78	67.25	35.34	22.20
Human+mouse	80.67	82.74	43.38	30.57
All 4 mammals	82.53	83.99	44.02	31.88
All 8 species	84.31	84.82	51.45	34.93
**100-ROC (%)**				
Human only	5.22	4.31	18.30	20.03
Human+mouse	2.45	1.93	13.18	15.54
All 4 mammals	2.21	1.81	11.77	14.75
All 8 species	1.76	1.54	10.53	11.68

The table shows the F score (geometric mean of sensitivity and specificity) and ROC error rate (area not under the ROC curve) for the horizontal component of Classifier Two trained on different numbers of informant species and running on the challenging evaluation (Eval) set. All scores are percentages.

**Table 5:** Exon and transcript accuracies

	DOGFISH-1	DOGFISH-2
Exon sensitivity	53.11	63.68
Exon specificity	77.34	84.90
Transcript sensitivity	5.08	8.94
Transcript specificity	14.61	33.12

The table shows percentage sensitivity and specificity at the exon and transcript levels for the workshop version, DOGFISH-1, and the current version, DOGFISH-2.
